# On Strength Variations Effected by Infill Patterns Such as Honeycomb, Gyroid, and Archimedean Chords Used in Additive Manufacturing

**DOI:** 10.3390/polym18131619

**Published:** 2026-06-29

**Authors:** Karolina Gocyk, Reza Afshar, Bilen Emek Abali

**Affiliations:** Ångström Laboratories, Division of Applied Mechanics, Department of Materials Science and Engineering, Uppsala University, P.O. Box 35, SE-751 03 Uppsala, Sweden; karogocyk@gmail.com (K.G.); reza.afshar@angstrom.uu.se (R.A.)

**Keywords:** 3D printing, additive manufacturing, polymers, infill pattern, infill density, gyroid, honeycomb, Archimedean chords

## Abstract

Additive manufacturing delivers internal substructures that alter the mechanical performance, yet their exploitation is still limited in structural part design, to a certain degree due to the absence of comparative studies. All slicer software solutions can exchange the infill with predefined infill patterns. Often their performance properties are unknown, and engineers make choices that depend on the printing time or material use. We conduct an experimental campaign to understand infill patterns’ effect on the mechanical performance. This work is inspired by biomimicry and studies honeycomb-, gyroid-, and Archimedean chords-type infill patterns in order to determine their performance. Experimental analysis via the three-point bending test has been conducted by using samples from PolyLactic Acid (PLA) with infill densities of 50, 60, 70, 80, 90 and 100% for these infill patterns. An additional set of samples was printed with Acrylonitrile Butadiene Styrene (ABS) for additional evaluation of Archimedean chords. We characterize the mechanical performance by comparing strength properties and observe that a mass-normalized flexural strength measure is meaningful when selecting an adequate infill pattern. Honeycomb showed the highest absolute flexural strength; strength per mass peaked at 90% infill. Mass reduction effected by infill density reduction fails to be linear; lowering infill down to 50% decreases mass marginally by up to 17% only. The performance of each infill pattern and comparisons between mass, strength, and print time are described to serve as a guide for designers.

## 1. Introduction

Additive Manufacturing (AM) is a rapidly evolving technology and operates with the highest design freedom among other manufacturing methods. This versatility has driven the expansion of this method to accommodate a wide range of materials, including metals, polymers, ceramics, and composite materials such as metal-infused polymer filaments [1,2,3,4]. The process involves depositing materials layer by layer to create the final product, which may require post-processing to achieve the desired mechanical or aesthetic properties [5,6]. Combination with more conventional techniques [7] and use in composite materials [8] may be seen as indicators of the future of the AM method.

One widely used additive manufacturing method is a Material EXtrusion (MEX)-based technique also called Fused Deposition Modeling (FDM) or Fused Filament Fabrication (FFF) [9], where a polymer filament—typically 1.7 mm thick—is extruded through a heated nozzle (orifice) in a molten form. As the nozzle moves with respect to the build plate, deposited material forms the product, gradually building up the part until completion. Therefore, all process parameters alter the product. The following parameters are empirically shown to be significant in material properties: printing temperature, ambient temperature, build plate temperature, molten material shape and form, extrusion speed, and acceleration [10,11]. Their role in mechanical properties has been investigated in [12,13,14,15], optimization techniques are generated a posteriori in [16,17] and even in operando conditions in [18,19,20].

One key novelty of AM lies in embodying an internal substructure to the CAD (Computer Aided Design) model by means of a so-called infill pattern that may be fine-tuned to fundamentally alter the mechanical response [21,22]. Effected by infill, materials exhibit multiscale features in statics [23,24] and dynamics [25,26,27]. Infill patterns are periodic shapes such that their geometry directly manipulates the material response [28,29,30], specifically with a periodicity at the same length-scale of the product itself [31,32,33]. Experimental studies exist in the literature for understanding how infill causes nonlinearity [34,35,36] or even damage propagation as in [37]. One of the widely used materials is PolyLactic Acid (PLA) in MEX-based 3D printers. Specifically in PLA, apart from material modifications as shown in [38,39,40], potential mechanical response alterations such as anisotropy rely on the infill pattern and density [41,42].

Similar to infill, internal substructures also exist in nature and are widely studied across academia for their mechanical performance. Those nature-inspired microstructures draw from biological sources like nacre’s brick-and-mortar layers [43,44,45], bone’s hierarchical porosity [46], honeycomb made by bees [47], gecko setae’s fibrillar arrays [48], and lotus leaf textures [49] in order to create engineered surfaces and materials with exceptional properties. These microscale architectures—often fabricated via additive manufacturing or nano-3D printing—enable combinations of high stiffness and toughness, lightweight design, reversible adhesion, superhydrophobicity, and stimuli-responsive behavior that bulk homogeneous materials cannot achieve. By mimicking evolution’s optimized templates from shells, spines, insect wings, and plant tissues, engineers develop robust components for aerospace, biomedical implants, self-cleaning coatings, and climbing robotics, decoupling performance from material composition for multifunctional efficiency.

Infill patterns are introduced by mimicking existing shapes in nature. Herein we study gyroid, which is a Triply Periodic Minimal Surface (TPMS) where gyroid crystals are witnessed in the wing scales of the papilionid butterfly Teinopalpus imperialis [50]. Composed by bees, the prominent hexagonal honeycomb structure is still a mystery [51]. Spiral shapes are often observed in microorganisms [52], and such an Archimedean chords infill has been used herein as well. Infill patterns are usually suggested to decrease the printing time and reduce the amount of material, yet often their role is neglected regarding mechanical properties. The infill acts as an internal framework within the printed part, providing a structural support. In other words, infill is a grid-like or truss-like pattern (substructure) inside the printed part that fills in the volume within the contours and walls of the printed part. Not only the infill density (also called infill ratio) but also the substructure (infill pattern or shape) modifies the mechanical performance. Indeed, due to imperfections in putting layers one on top of the other, even the 100% infill (bulk specimen) contains pores and is lighter than an injection molding. This porosity causes a decrease in mechanical performance as well, yet it is different from the structural performance altered by the internal substructure. The choice of infill pattern and ratio is critical for determining the durability and mechanical properties of the final printed product.

In this work, we analyze the effect of the chosen infill patterns on the strength of the part. By following biomimicry, three nature-inspired infill patterns, namely honeycomb, gyroid, and Archimedean chords, have been examined with infill percentage ranging from 50% to 100%. This range is technically relevant and has allowed us to create a quantified evaluation of each infill’s mechanical performance. The robustness of infills’ mechanical support has been evaluated by conducting a three-point flexural test that investigates the increase of bending rigidity regarding infill pattern and percentage. Flexural strength has been calculated and tabulated in order to be used in part design by incorporating the infill pattern. We simplify the analysis by discarding several effects involving multiscale, nonlinear, and dissipative modeling. After explaining the used methods in Section 2, we discuss the results in Section 3, and then sum up in Section 4.

## 2. Materials and Methods

Samples were printed by using a Prusa i3 MK3S+ MEX printer, Prusa Research, Prague, Czechia. The material used for fabrication was PLA, using E-PLA filament—Easy PLA is made out of the bio-degradeable bio-plastics PLA by Add:North, Sweden. According to the manufacturer, the filament has a nominal diameter of 1.75 ± 0.02 mm, a melting temperature range of 195–225 °C, and a recommended printing temperature of 205–225 °C for the nozzle and 0–60 °C for the bed. The material was stored in a dry environment prior to use to minimize moisture uptake. For the printing, a nozzle temperature of 210 °C and a bed temperature of 60 °C were set. All print parameters are compiled in Table 1 and the G-code is generated by Bambu Studio slicer software version 01.08.04.51.

The nozzle temperature of 210 °C was chosen as a compromise within the recommended range of 205–225 °C for this PLA, balancing filament flow stability, dimensional accuracy, and avoidance of overheating or stringing. Temperatures closer to 205 °C can lead to under-extrusion and weak interlayer bonding, whereas temperatures above approximately 220 °C increase the risk of material degradation and reduced surface quality; thus, 210 °C was selected as a stable mid-range setting for all tests to isolate the effect of infill pattern rather than temperature. Bed temperature was selected with similar compromise to optimize the bed adhesion. Higher temperatures loosened the grip on the plate and the print started moving freely. Layer height of 0.2 mm was chosen as an engineering standard for 0.4 mm nozzle in order to optimize the print time versus strength. Lower layer height, say 0.15 mm, would potentially increase strength due to increased interlayer, while using a lower layer height, say 0.3 mm, would speed up the manufacturing process. The same nozzle and layer height was used for all specimen.

**Figure 1:**
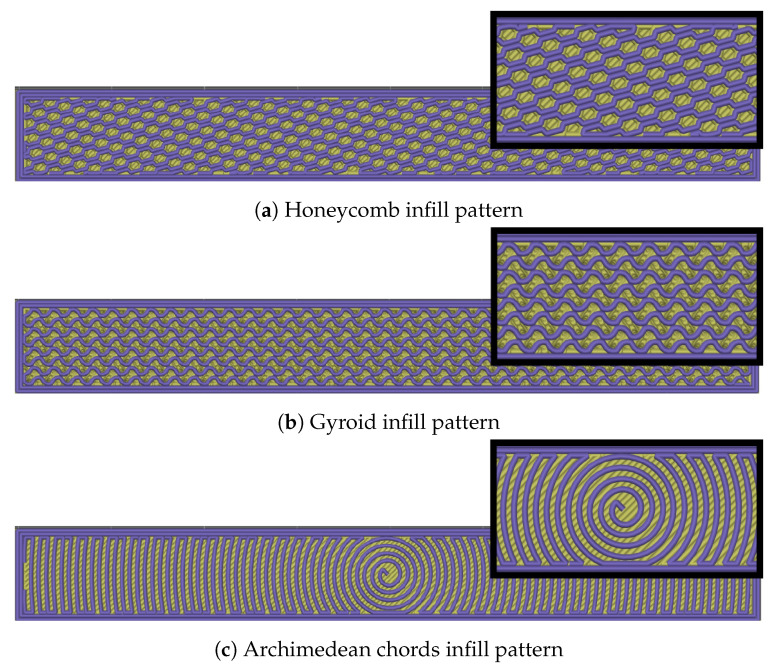
Illustration of different infill patterns from slicer software in a top view. An additional inset is placed for the sake of visualization. Outer walls are full material such that the views are generated in a cut.

By using PLA, three sets of samples were manufactured with different infill patterns, namely gyroid, honeycomb, and Archimedean chords, as depicted in Figure 1. Each infill pattern was printed with infill density of 50%, 60%, 70%, 80%, and 90%. Three samples were printed for each infill density. For 70% infill density, five samples were printed in order to quantify manufacturing repeatability. Additionally, five samples of full bulk filament (100% infill) were prepared as a control group for the experiment. After the printing, samples were conditioned for 24 h in a stable temperature at 40 °C (in a dryer) to remove any moisture content. We follow ASTM5229 [53] and approximate the diffusion as Fick’s law where a closed-form solution may be approximated as $h=\sqrt{4Dt}$ and approximate $D=1\times 10^{-9}$ m^2^/s as the water diffusion in poymers such as PLA. Therefore, 24 h conditioning should bring a specimen up to h=18.5 mm to the same environmental humidity value. We stress that the specimens are around 4 mm thick. All tests were performed in an as-printed condition without further post-processing or annealing.

By using ABS, a new set of specimens was printed with Archimedean chords for increasing the understanding of the infill pattern effects. In total, infill densities 70%, 75%, 80%, 85% and 90% in were printed in the quantity of 5 for each infill density. The choice is for a more in-depth discussion of structural effects near the 80% infill density. Open enclosure printing setup results in unreliable warpage for ABS since the material is semi-crystalline and shrinks in anisotropic manner leading to a warpage or even releasing the board during printing. Therefore, ABS samples were printed on Bambu X1 Carbon MEX 3D printer with parameters compiled in Table 2.

**Figure 2:**
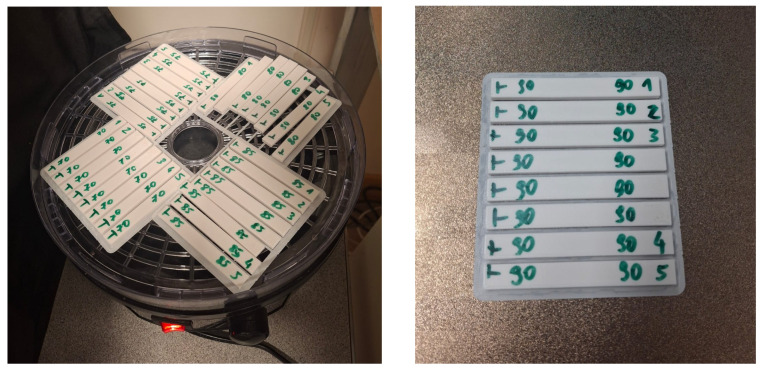
Printed ABS samples (**left**)—drying, (**right**)—on the print plate.

All geometric dimensions were kept the same as in the case of PLA; however, ABS results in a significant shrinkage—for example, the thickness was 4 mm instead of 4.3 mm. In order to compensate such differences in all calculations, as-printed dimensions were used by measuring them for each specimen. All the samples were dried and conditioned similarly to PLA prior to testing, see Figure 2.

**Figure 3:**
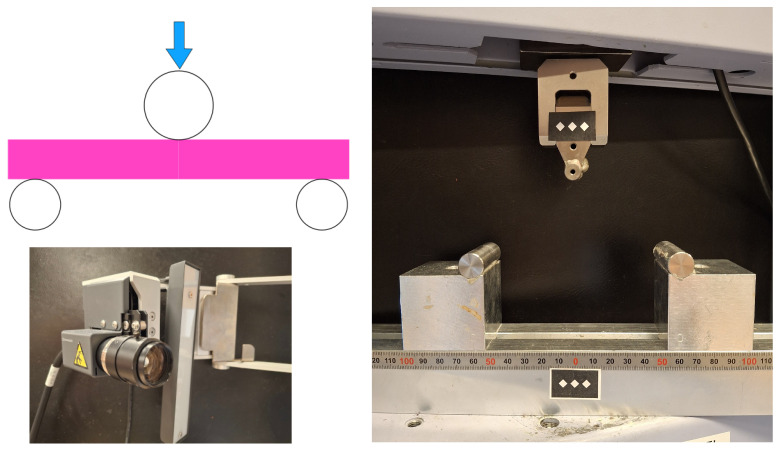
(**Left top**): Schematic depiction of a three-point flexural test setup; on two supports at the bottom, a (pink) beam is placed that is loaded by the (blue) arrow in vertical direction. This setup creates a normal strain/stress response that is negative on the top and positive on the bottom end creating a bending motion. (**Left bottom**): Optical (contactless) extensometer to track the displacement of markers. (**Right**): Three-point bending jig test setup in the laboratory.

In order to investigate the strength under bending, three-point (flexural) tests were performed as shown in Figure 3 by following the European Norm PN-EN ISO 178 [54].

The sample dimensions were modeled to be 80×10×4 mm (length × width × height), but due to material deposition, the final dimensions were 80.0×10.0×4.3 mm. All tests were carried out using a Shimadzu Autograph AGS-X uniaxial tensile/compression machine with a loadcell of 10 kN of ±1% precision. During the test, samples were placed on an in-house designed and manufactured jig with roller-type supports on a 60 mm span distance. Displacement was measured by using an integrated camera system tracking a printed-out marker placed on the top part of the jig. The test was conducted with speed 2 mm/min. The force and displacement data from the tests were converted to flexural stress and strain using standard beam bending equations. In the case of three-point beam bending, stress, σ, and strain, ε, are calculated from the measured force, *F*, and displacement/deflection, δ, as follows:

(1) $$\sigma =\frac{3FL}{2wt^{2}}\;,\;\varepsilon =\frac{6t\delta }{L^{2}}\;,$$

where the support span, L=60 mm, the specimen width w=10 mm, and the thickness, t=4 mm, are used for all specimen. As initial geometric dimensions are used in the stress/strain calculation, the obtained values are engineering (Piola) stress and engineering strain. We emphasize that the infill density is discarded in this calculation, meaning that strength reduction is expected naturally by distributing the force to a smaller area as the infill density decreases. This interpretation is correct as long as the infill pattern characteristic length is small regarding the geometric dimensions. More directly, infill pattern is chosen in the slicer, and the spatially repeating pattern has a unit cell length. Depending on the used slicer software this unit cell length is set and often it is in the same order of magnitude as the geometry. Therefore, despite the fact that we use Equation (1), we also remark that an infill pattern alters the material properties, and this phenomenon may be modeled by higher-gradient theories [55]. For the sake of simplicity, with a grain of salt, we are going to apply Equation (1) under the assumption that material parameters change depending only on the porosity but not on the structure itself.

## 3. Results and Discussion

**Figure 4:**
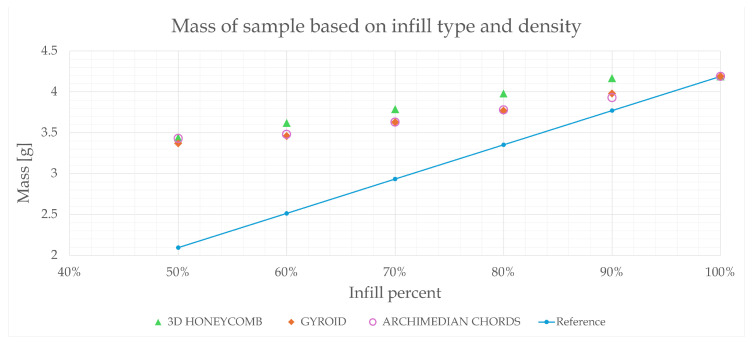
Expected mass as a reference line and measured mass for each infill pattern as points.

Strength of material is the load bearing capacity related to the cross sectional area under load, depending on the structure. A direct analogy is that a porous structure with increasing porosity has less area, and thus, we expect less strength. We may introduce mass and relate strength to mass, which is indeed a result of a homogenization procedure of the first order by assuming that a porous structure is a mixture of material and air, where strength of air is null and material carries linearly scaled to the one minus porosity. This approximation is a first-order theory since the substructure is neglected by the homogenization approach. This assumption leads to the well known mass-normalized flexural strength also used as a normalized measure in materials charts [56]. Therefore, we enlist specimen weights in Table 3 and plot them in Figure 4 in order to visualize the deviation of the manufacturing result from the expected mass with the given infill percent.

Mass was measured by a laboratory scale with a precision of ±0.1 mg up to a maximum capacity of 220 g, with a minimal load of 10 mg and repeatability precision of ±0.08 mg. In many cases, the infill density is taken as the bulk, say, 80% infill is analyzed with 80% of modulus and strength. The reference line in Figure 4 symbolizes exactly this choice, namely, the literal percentage of bulk (maximal) mass of 4.19 g, so that 50% is precisely half of 4.19 g, 60% is 0.6×4.19 g, and so on. However, this assumption is misleading in two ways. First, we realize in Figure 4 that the mass distribution fails to be the same as the infill percentage. The printing strategy and the infill pattern alter the outcome. There are different factors for this deviation. Walls are used for creating a shell, and walls are printed as full material, increasing the overall mass. Also, the slicer algorithm creates the substructure of choice with a given infill density target, yet, it is an inexact method to maximize the printability. Second, the infill pattern effects are present, and they are discussed in detail in the following.

From the mass weighting procedure, we understand that honeycomb infill has a significantly higher mass than other two infill patterns in each percentage. This difference indicates that despite theoretically the same amount of filled-in volume, gyroid and Archimedean infill patterns harbor more porosity, therefore making the supportive scaffold of the print with less PLA. In order to calculate the actual porosity, 4.19 g was assumed to be a true 100% bulk sample. The mass percentages of samples are shown in Table 3.

It is important to note that all of the infill patterns and ratios vary between 80 and 100% mass, which indicates that by decreasing infill by 50%, it is only possible to reduce approximately 20% of mass. On one hand, skipping the step of measuring the actual mass and instead extrapolating from an experimental 50% infill value will overestimate the material properties. On the other hand, a 50% infill may alarm designers, but the material properties are often better than 50% of the original value—ignoring buckling and manufacturing defects—which explains the widespread use of low infill densities in practice. We also emphasize that the knowledge of this fact is important, since the slicer algorithms may change in future, leading to a decrease of the strength that is not meeting expectations. One possible outcome is that designers may know the discrepancy and design with higher infill ratios to mitigate such problems.

**Figure 5:**
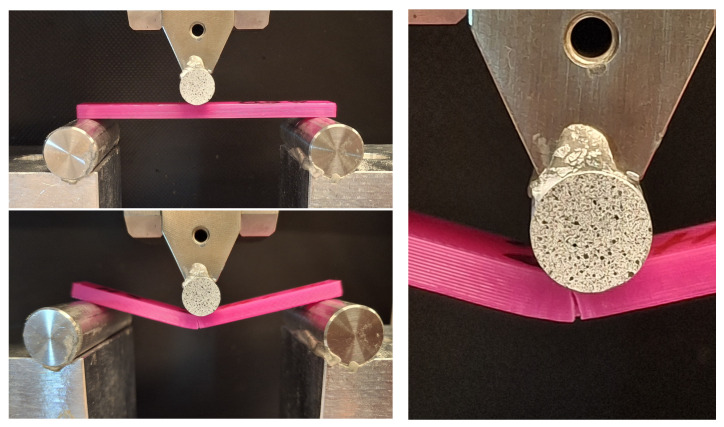
(**Upper left**): Sample placed on the machine supports before breaking. (**Bottom left**): sample on the machine after breaking (in this case it is one of Honeycomb infill samples). On the (**right**): A close-up view of the broken specimen. In all experiments the top surface (5 layers thick) faces upwards, being marked, as it is the surface where sample labeling with a permanent marker was done.

During the experimental procedure, all specimens were placed on the machine supports so that the top of the sample, which is the surface mechanically loaded, had 5 layers (see Figure 5). Those layers were made with 100% monotonic line infill, which consists of closely aligned lines of extruded material. This setup allows a constant bending stress (that is the normal stress applied on the extruded material lines) across the cross sectional area.

**Figure 6:**
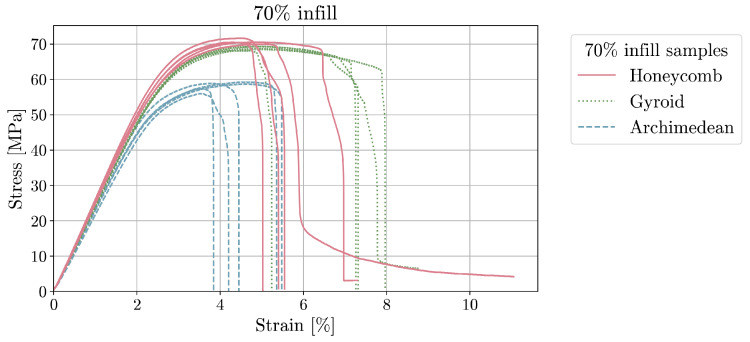
Three-point flexural results for 70 % infill.

In order to test the repeatability in additive manufacturing, we perform a test with the 70%-infill specimens in Figure 6. All infill patterns were printed with 5 specimens at once and tested to attest for the repeatability of the manufacturing method. We note by a visual inspection of results in Figure 6, the values of which present an insignificant scatter. For quantifying the repeatability, an arithmetic mean value for each infill type is calculated by giving the standard deviation as follows:

(2) $$\begin{array}{c} s=\sqrt{\frac{1}{n-1}\sum_{i=1}^{n}{\left(x_{i}-\bar{x}\right)}^{2}}\;, \end{array}$$

where $x_{i}$ are the individual data points, $\bar{x}=\sum_{i}x_{i}/n$ is the arithmetic mean, and *n* is the number of data points. The latter measures the typical deviation of data points from the mean value, quantifying the spread or variability in the data. It is widely used in experimental data analysis to express the precision of measurements. The results are compiled in Table 4.

We observe a relatively small standard deviation, roughly around 1%, for honeycomb and gyroid patterns, leading to a 95% confidence level for the sample size 5. However, Archimedean infill pattern performs worse signaling that there is a need of a better understanding why this infill pattern has more challenges to provide the repeatability, which is given for other infill patterns.

**Figure 7:**
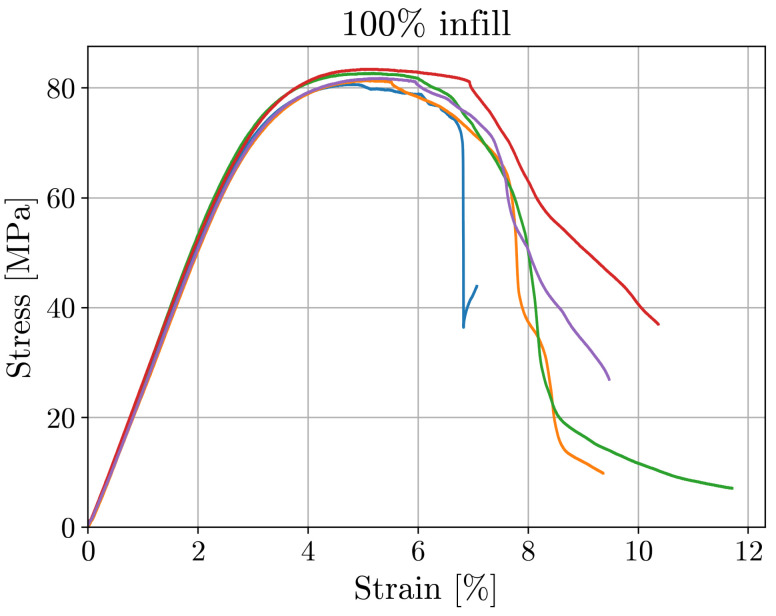
Three-point flexural results for 100% infill density samples.

The bulk specimens were printed out and tested as a reference gauge for evaluating infill patterns in Figure 7. As expected, these samples perform with the highest (average) strength of 81.93 MPa as given in Table 6. This result is aligned with an educated guess that 100% is the best mechanical performance since they have more material in their structure. We emphasize that the bulk sample is filled in completely with material, thus, there is actually no infill pattern introduced for a full specimen. Indeed, there is some porosity because of the layer-by-layer production in additive manufacturing. In MEX based printing, a round nozzle leads to a closely packed layers structure.

**Figure 8:**
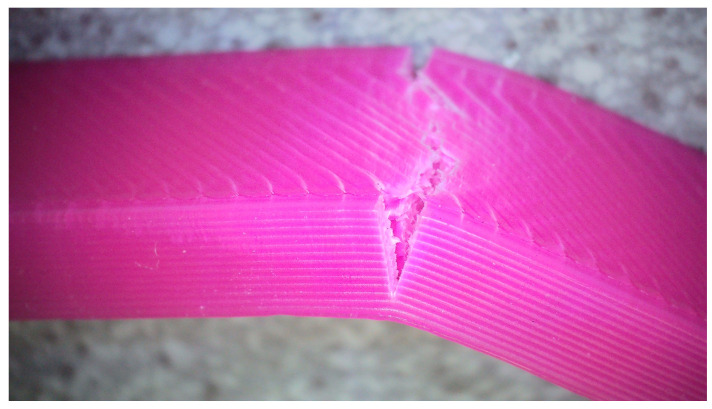
Three-point beam bending until failure, in the case of 100% infill, as seen under a light microscope showing the typical mode I fracture with some deviations due to printing irregularities.

The fracture under three-point beam bending is mode I as seen in Figure 8. We stress that the (brittle) fracture surface is flat in wall sections, where the printing speed is lower as expected from the bulk material. However, infill region with 100% infill density shows some irregularities directly related to manufacturing imperfections leading to varying porosity along the thickness.

**Figure 9:**
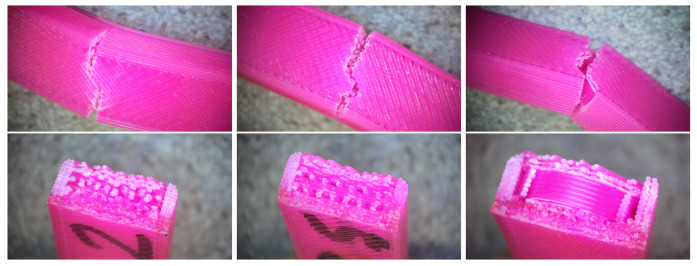
Three-point bending beam until failure for (**left** to **right**) honeycomb, gyroid, Archimedean infill patterns, (**top**): mode I crack formation, (**bottom**): the corresponding fracture surface.

This porosity value and its distribution is discarded herein and the print is understood as the bulk material with 100% infill. Similar fracture surface and deviations from mode I crack formation has been identified in other infill patterns as well as depicted in Figure 9.

The observed deviations from an ideal mode I fracture are likely associated with the geometry-dependent stress distribution and inter-road bonding characteristic of the different infill structures. In particular, infill patterns with continuous load paths are expected to promote more uniform crack propagation, whereas patterns containing frequent directional changes or discontinuous interfaces may lead to localized stress concentrations, crack deflection, and partial interlayer delamination. Furthermore, manufacturing-induced porosity and variations in filament fusion may contribute to irregular fracture surfaces and non-uniform crack growth behavior under bending loads. Therefore, an analysis seems to be difficult for fracture toughness and critical release rate. We limit the scope to ultimate tensile strength and skip a discussion about the energy release during crack propagation, we refer to [57] for a similar analysis.

The (average) mass of bulk samples is 4.19 g, to which the closest is honeycomb 90% with mass of 4.17 g as seen in Figure 4.

**Figure 10:**
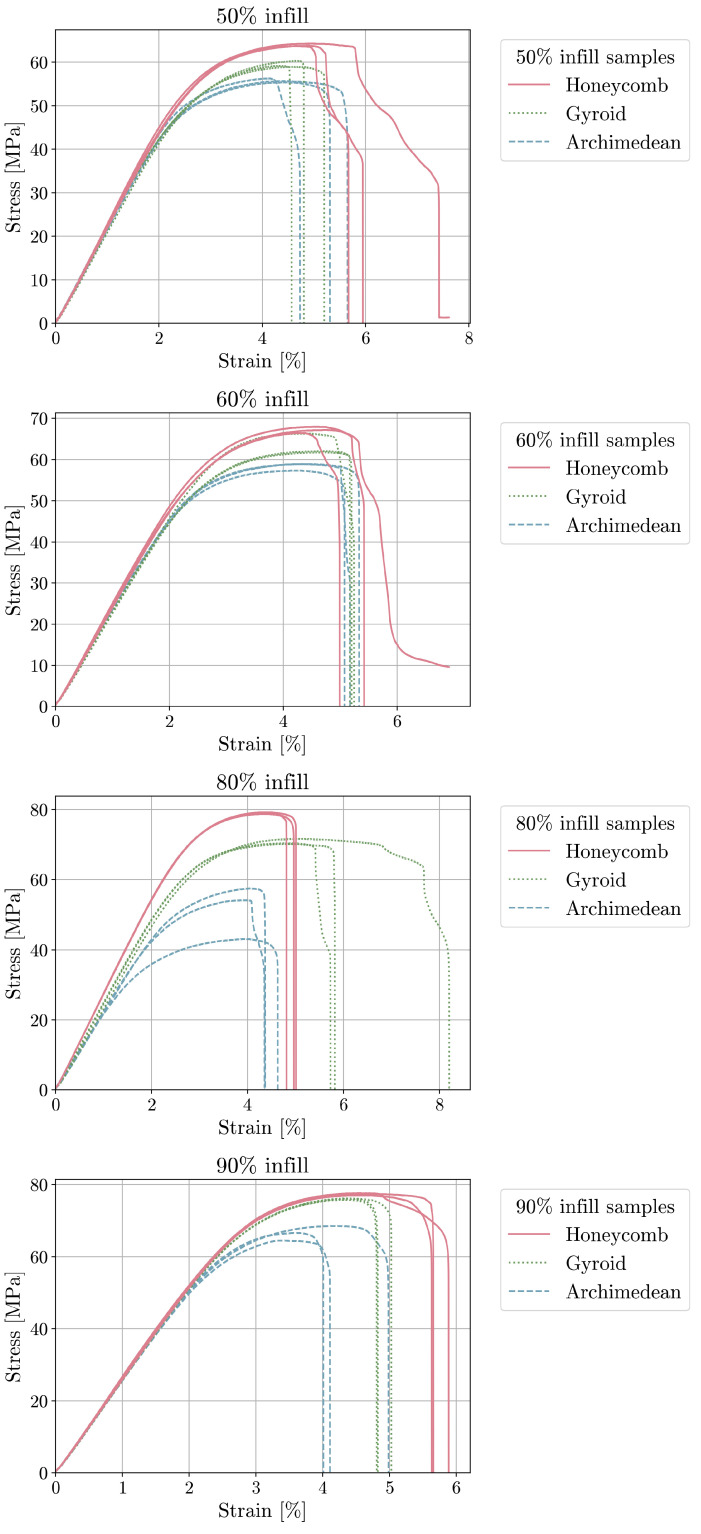
Three-point flexural results 50%, 60%, 80%, 90% infill densities.

At lower infill densities, we observe differences in mechanical strength among the infill patterns. The honeycomb infill pattern demonstrated the highest mechanical strength, with an average strength of 64 MPa at 50% infill and 67 MPa at 60% infill in Figure 10. In contrast, the Archimedean and gyroid infill patterns showed similar performance, with lower strength values and similar graph layouts.

For infill percentages between 70% and 90%, the results differed. The performance gap between Archimedean and gyroid infill patterns became more pronounced, and a noticeable difference in strengths and graph layouts emerged between these two patterns. We emphasize the negligible mass difference for 70% infill density between Archimedean and gyroid patterns in Table 3. However, strength values present a stark distinction between Archimedean and gyroid infill patterns and clearly depict the gyroid as stronger. This effect is structural, caused by the geometry (infill pattern). Moreover, we stress that these substructures introduce an anisotropy to the specimen such that their orientation along the normal stress direction (along the beam axis) makes the infill pattern more favorable in the conducted flexural tests. This interpretation is even more general and needs to be modeled by higher gradient models that is out of scope in this study. With respective masses of 3.79 g for honeycomb and 3.63 g for Archimedean and gyroid, the honeycomb pattern exhibits a strength of 71 MPa and gyroid a strength of 69 MPa, while Archimedean achieves a strength of 58 MPa. This significant difference in mechanical performance is substructure related, since the average mass of Archimedean and gyroid remains the same.

For quantifying the manufacturing and measurement errors, samples with 70% infill were printed in larger quantities (five specimens). Strength variation is relatively small for all patterns as compiled in Table 4. This consistency supports the reliability of conclusions drawn from fewer samples in the rest of the experiments. With 70% and 80%, the gyroid infill has significantly larger deformation than the honeycomb infill pattern, while in other percentages this tendency is reversed. In the case of 80%, the honeycomb pattern has the highest strength of 79 MPa from Figure 10 as given in Table 5.

In the case of the 90% infills as in Figure 10 all the graphs come closer to each other, signifying that the choice of substructure becomes less relevant by converging to 100% bulk. Gyroid and honeycomb are again close to each other in terms of strength with respective results of 76 MPa and 77 MPa as given in Table 6. Archimedean chords are the weakest with a strength of 67 MPa on average. The infill density of 80% has a significantly lower performance than the trend seen in Table 6. The 90% infill proves to have the best mechanical parameters in Figure 10 while we stress the large strain of 6% to 7% in the case of 50% and 60% infills Figure 10.

In order to eliminate the influence of different density and therefore mass on specimens’ durability and mechanical performance, all average results for each infill pattern are divided by their respective average mass in Figure 4, resulting in values expressed in MPa/g in Table 7.

Table 7 shows that the honeycomb infill generally exhibits the highest performance, whereas the Archimedean chords pattern tends to perform the worst. A noteworthy exception occurs at 80% infill, where the performance of the Archimedean chords pattern drops sharply, while that of the honeycomb pattern increases markedly. This behavior is investigated further in what follows by focusing around 80% infill density as well as performing the experiments with ABS.

**Figure 11:**
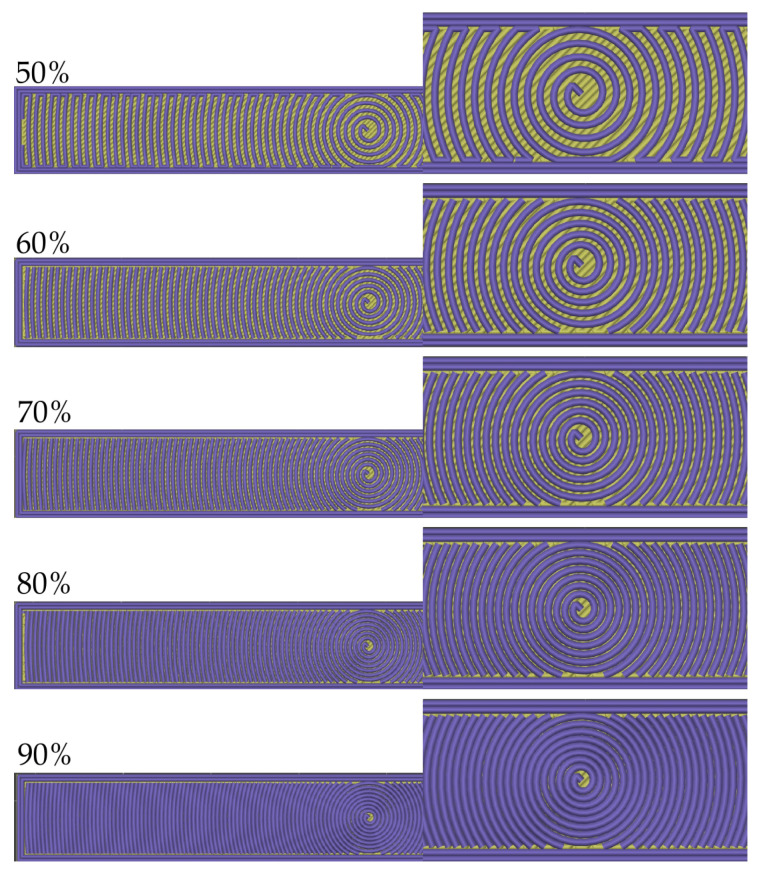
Visual analysis of the Archimedean chords infill.

**Figure 12:**
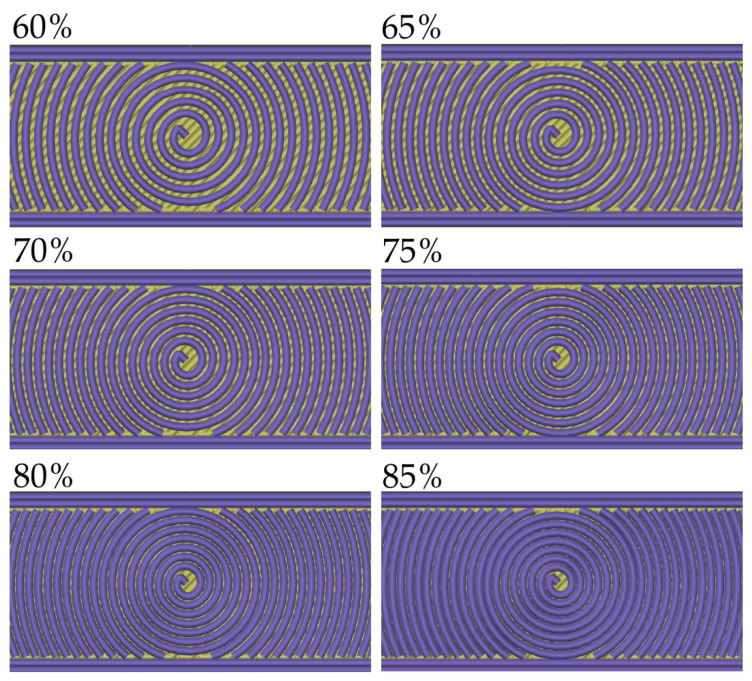
A close-up look at the middle of Archimedean chords infill pattern with infill % closer to 80%.

We investigate the structure introduced by Archimedean chords. Figure 11 demonstrates slicer visualization for 50%, 60%, and 70% infill densities, obviously, the middle circle is smaller than for 80%. In 50% most of the spiral is a continuous line providing an adequate support, but significant gaps make it prone to deformation without contact between the lines and also breaking along the wider gaps. Oppositely at 90%, lines are dense that they have a tendency to melt into each other while being deposited by an extruder, which brings a 90% infill very close to a full bulk sample significantly improving the strength. So 80% seems to generate such a geometry where the middle spiral is large in size, creating one unsupported part, while the lines drawn by extruder are not close enough to meet and melt into each other. Furthermore, with the internal structure made of arced lines neighboring each other in a way seen in Figure 11 and Figure 12, it is noted that each of them is a potential weak point, analogous to abovementioned discussions for 50%. This structural effect could be that 80% Archimedean chords have several (more than infill densities that came before) relatively weakly connected filament lines creating the potential breaking points. The main issue is that one line is not enough to create load carrying structures.

For testing this hypothesis, we use ABS and perform additional tests with a denser choice of infill density variation around 80%. Specifically, 70%, 75%, 80%, 85%, and 90% infill densities were manufactured (each with 5 samples) and tested as shown in Figure 12.

**Figure 13:**
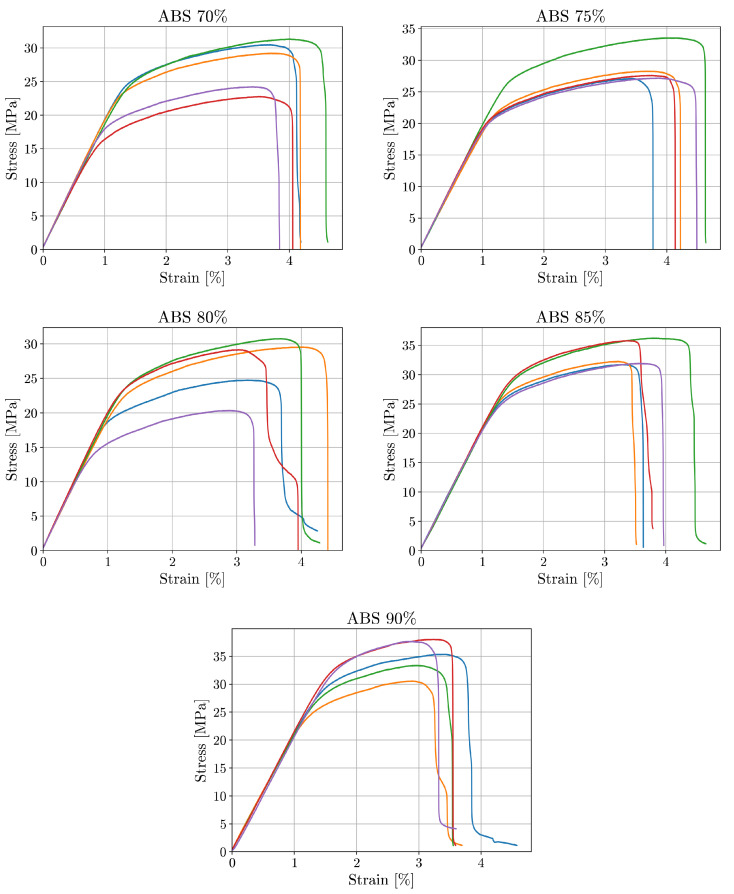
Results in the form of stress–strain graphs for ABS printed samples of Archimedean chords with infill density ranging from 70% to 90%, each infill density has been tested with 5 samples indicated as different colors.

Results are presented in Figure 13 and in Table 8 and they are in line with the observed anomaly obtained for PLA samples. The same type of strength drop around 80% is visible also with this new set of specimens and a different material. Archimedean chords infill lacks a structural support for this geometry specifically around 80% infill density. As it may be observed visually in Figure 12 that the 80% performs weaker than 75%. On the contrary, 85% should come closer to the performance of 90% infill density.

Already indicated by the repeatability test, here with ABS, the same observation is possible that 80% infill density performs not only weaker but also has a higher variation between each print. The structure is the reason for this. Indeed the choice of 80% is accidentally performing weak for the chosen specimen thickness. For 80% there seems to be a lot of breaking points vulnerable for three-point bending test as well as not as much inter-line melting of polymer lines needed for an improved cohesion, which happens at higher infill densities of 85% and 90%. In general, Archimedean chords may be risky to use in bending related loading scenarios.

## 4. Conclusions

Across this study the honeycomb infill tends to exhibit higher (flexural) strength, compared to gyroid and Archimedean chords, especially at lower infill densities (50% and 60%). Gyroid infill consistently demonstrates a high strength ratio across all infill percentages, often approaching or exceeding the strength of honeycomb infill, particularly at higher infill densities (above 60%). Archimedean chords infill shows the weakest mechanical performance compared to the other two patterns across most infill densities. At 70% infill, a significant performance gap becomes evident between gyroid and Archimedean chords. At 80% infill density, Archimedean chords also demonstrate a noticeable reduction in flexural strength. This behavior is consistently observed in the investigated specimens and suggests a geometry-dependent structural effect related to the load-bearing architecture of the infill pattern.

Previous work by [58], conducted under comparable ISO-178 three-point bending conditions using rectilinear infill, reported flexural strengths of 119.66 MPa at 70% infill density, 110.66 MPa at 50% infill density, and 102.00 MPa at 30% infill density. In the present study, the measured values for the investigated infill patterns at 70% infill density ranged from 58.01 to 70.60 MPa, while at 50% infill density values ranged from 55.77 to 63.91 MPa. Although direct quantitative comparison between studies remains difficult due to differences in the chosen infill pattern, PLA filament brand (we stress that the PLA is a mixture of PLLA and PDLA that alters the strength), and process parameter selection; both studies indicate that infill pattern and density significantly influence flexural performance under bending-dominated loading conditions. We have simplified the analysis by simplifying the relations with strength per porosity (infill density) and ignoring infill pattern related higher grade effects altering material parameters.

We focus herein on the infill pattern and density but also stress that the process parameters have an effect, too. Material parameters are altered not only by the printing parameters and material composition but even by the slicer strategy and post-processing conditions. Variations in printer type, nozzle condition, thermal control, raster generation, and filament manufacturer may significantly influence the resulting mechanical properties, which complicates direct quantitative comparison between independent studies. Consequently, investigations of infill topology and mechanical characterization should primarily be interpreted as comparative studies within a controlled experimental framework. Increasing the infill density generally improves the mechanical properties of the samples, as expected. Specifically, 90% infill demonstrates the best mechanical parameters among the tested infill densities. We remark that the finding is limited by the specific wall settings, herein, we use a full dense shell and top and bottom layers. By increasing the thickness, the quantitative results will change but the overall tendency remains the same that we need a design parameter normalizing the mass.

The results additionally indicate that infill density impacts the final prints porosity. Honeycomb infill results in parts with a higher mass, suggesting lower porosity compared to gyroid and Archimedean chords at the same infill density. This tendency indicates that gyroid and Archimedean infills create more internal voids, using less material for the supporting structure. In general, infill is introduced to optimize the material use as well as print time. Decreasing the infill density by 50% only reduces the mass of the printed part by approximately 20%, which indicates that there is a limited impact of infill reduction on mass savings and this factor should be taken into consideration in the case of topology optimization in structural mechanics.

**Figure 14:**
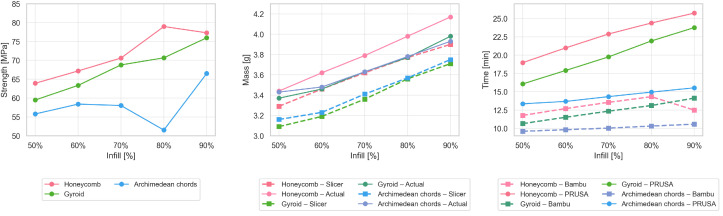
Comparison of all important investigated parameters as a guide for manufacturing.

Viewing a summary of performance at Figure 14, Archimedean chords excel at low mass and fast printing times, while honeycomb displays the highest flexural strength at a cost of highest mass. Gyroid infill demonstrated the most balanced trade-off between flexural strength, mass efficiency, and printing performance across the investigated infill densities. A possible comparison is of course strength per mass such that the topology optimization and print time are both merged in one measure.

The conclusions are limited to the investigated printer settings, loading orientation, nozzle size, and manufacturing conditions. Different slicers, raster orientations, or processing conditions may alter the quantitative trends. 

## Figures and Tables

**Table 1:** Print parameters used for all infill patterns to print with PLA.

Parameter	Value
Infill pattern	Gyroid, honeycomb, Archimedean chords
Infill density	50–100%
Bed temperature	60 °C
Nozzle temperature	210 °C
Layer height	0.2 mm
Cooling/Fan speed	70%
No cooling	For the first layer
Seam position	Aligned
Wall loops	2
Top/bottom shells	Monotonic line
Number of top shell layers	5
Number of bottom shell layers	3
Supports	None
Skirt loops	None
Brim	None
Rib wall	None

**Table 2:** Print parameters used for all infill patterns to print with ABS.

Parameter	Value
Infill pattern	Archimedean chords
Infill density	70–90%
Bed temperature	90 °C—textured PEI/High temp. plate
Nozzle temperature	270 °C
Layer height	0.2 mm
Cooling/Fan speed	60%
No cooling	For the first layer
Seam position	Aligned
Wall loops	2
Top/bottom shells	Monotonic line
Number of top shell layers	5
Number of bottom shell layers	3
Supports	None
Skirt loops	None
Brim	Outer brim only: 5 mm
Rib wall	None

**Table 3:** Measured mass of specimens (arithmetic average) given as mass percentage to the 100% bulk sample.

Infill Percentage (Expected Value)	Honeycomb	Gyroid	Archimedean Chords
50%	$3.44\;g\;\hat{=}\;82.10\%$	$3.37\;g\;\hat{=}\;80.43\%$	$3.43\;g\;\hat{=}\;81.86\%$
60%	$3.62\;g\;\hat{=}\;86.40\%$	$3.46\;g\;\hat{=}\;82.58\%$	$3.48\;g\;\hat{=}\;83.05\%$
70%	$3.79\;g\;\hat{=}\;90.45\%$	$3.63\;g\;\hat{=}\;86.63\%$	$3.63\;g\;\hat{=}\;86.63\%$
80%	$3.98\;g\;\hat{=}\;94.99\%$	$3.77\;g\;\hat{=}\;89.98\%$	$3.78\;g\;\hat{=}\;90.21\%$
90%	$4.17\;g\;\hat{=}\;99.52\%$	$3.98\;g\;\hat{=}\;94.99\%$	$3.93\;g\;\hat{=}\;93.79\%$
100%	$4.19\;g\;\hat{=}\;100\%$	$4.19\;g\;\hat{=}\;100\%$	$4.19\;g\;\hat{=}\;100\%$

**Table 4:** Experimental mean values of flexural strength for 70% infill density.

Pattern	Strength [MPa]
Honeycomb	
Honeycomb 1	70.54
Honeycomb 2	69.92
Honeycomb 3	71.63
Honeycomb 4	70.38
Honeycomb 5	70.51
Mean ± Std. Dev.	70.60 ± 0.63
Gyroid	
Gyroid 1	69.32
Gyroid 2	68.45
Gyroid 3	69.28
Gyroid 4	68.73
Gyroid 5	68.11
Mean ± Std. Dev.	68.78 ± 0.53
Archimedean Chords	
Archimedean Chords 1	56.00
Archimedean Chords 2	57.37
Archimedean Chords 3	58.87
Archimedean Chords 4	58.63
Archimedean Chords 5	59.19
Mean ± Std. Dev.	58.01 ± 1.32

**Table 5:** Experimental mean values of flexural strength for 50%, 60%, 80%, 90% infill densities.

Pattern	Strength [MPa]
50% infill	
Honeycomb 1	63.77
Honeycomb 2	64.27
Honeycomb 3	63.68
Gyroid 1	59.16
Gyroid 2	60.30
Gyroid 3	58.94
Archimedean Chords 1	56.27
Archimedean Chords 2	55.67
Archimedean Chords 3	55.38
60% infill	
Honeycomb 1	66.41
Honeycomb 2	67.95
Honeycomb 3	67.18
Gyroid 1	66.30
Gyroid 2	61.73
Gyroid 3	62.01
Archimedean Chords 1	57.31
Archimedean Chords 2	58.91
Archimedean Chords 3	58.91
80% infill	
Honeycomb 1	79.08
Honeycomb 2	78.66
Honeycomb 3	79.15
Gyroid 1	71.58
Gyroid 2	70.10
Gyroid 3	70.30
Archimedean Chords 1	43.05
Archimedean Chords 2	57.40
Archimedean Chords 3	54.04
90% infill	
Honeycomb 1	76.99
Honeycomb 2	77.37
Honeycomb 3	77.58
Gyroid 1	75.76
Gyroid 2	76.14
Gyroid 3	76.05
Archimedean Chords 1	64.49
Archimedean Chords 2	66.58
Archimedean Chords 3	68.47

**Table 6:** Flexural strength comparison using averages.

Pattern	Expected Density [%]	Measured Density (from Mass) [%]	Average Strength [MPa]	Regarding Bulk
Honeycomb	50%	82%	63.91	78%
	60%	86%	67.18	82%
	70%	90%	70.60	86%
	80%	95%	78.96	96%
	90%	100%	77.31	94%
Gyroid	50%	80%	59.47	73%
	60%	83%	63.35	77%
	70%	87%	68.78	84%
	80%	90%	70.66	86%
	90%	95%	75.98	93%
Archimedean	50%	82%	55.77	68%
	60%	83%	58.38	71%
	70%	87%	58.01	71%
	80%	90%	51.50	63%
	90%	94%	66.51	81%
Bulk	–	–	81.93	100%

**Table 7:** Mass-normalized flexural strength results by dividing average flexural strength by measured mass for different infill patterns.

Slicer Infill	Honeycomb	Gyroid	Archimedean Chords
50%	18.58	17.65	16.26
60%	18.56	18.31	16.77
70%	18.63	18.95	15.98
80%	19.84	18.74	13.62
90%	18.54	19.09	16.93
Bulk	19.56	19.56	19.56

**Table 8:** Strength mean values for ABS specimen with Archimedean chords infill pattern, 5 specimen for each infill density as shown in Figure 13.

Infill Density	Flexural Strength
70%	27.60
75%	28.73
80%	26.91
85%	33.57
90%	34.98

## Data Availability

The original contributions presented in this study are included in the article. Further inquiries can be directed to the corresponding author.

## References

[1] Masood S.H., Song W.Q. (2004). Development of new metal/polymer materials for rapid tooling using fused deposition modelling. Mater. Des..

[2] Karimi N., Fayazfar H. (2024). Sustainable metal-infused polymer feedstock compatible with low-cost metal sinter-based 3d printing. Trans. Can. Soc. Mech. Eng..

[3] Chen C., Huang B., Liu Z., Chen L., Li Y., Zou D., Chang Y., Cheng X., Zhou R., Liu Y. (2024). Material extrusion additive manufacturing of WC-9Co cemented carbide. Addit. Manuf..

[4] Kalia K., Ameli A. (2024). Additive manufacturing of functionally graded foams: Material extrusion process design, part design, and mechanical testing. Addit. Manuf..

[5] Kantaros A., Ganetsos T., Petrescu F.I.T., Ungureanu L.M., Munteanu I.S. (2024). Post-production finishing processes utilized in 3D printing technologies. Processes.

[6] Isidore Besong L., Buhl J. (2024). A review of constitutive models used in macroscale finite element analysis of additive manufacturing and post-processing of additively manufactured components. Virtual Phys. Prototyp..

[7] Alzyod H., Kónya G., Ficzere P. (2025). Integrating additive and subtractive manufacturing to optimize surface quality of MEX parts. Results Eng..

[8] Guo P., Wu J., An X., Zhou Z., Yang D., Zhang H. (2025). On the mitigation of the fiber breakage in material extrusion based additive manufacturing of carbon fiber reinforced polymer composites. Addit. Manuf..

[9] Prabhakar M.M., Saravanan A., Lenin A.H., Mayandi K., Ramalingam P.S. (2021). A short review on 3D printing methods, process parameters and materials. Mater. Today Proc..

[10] Hasan M.R., Davies I.J., Pramanik A., John M., Biswas W.K. (2024). Impact of process parameters on improving the performance of 3D printed recycled polylactic acid (rPLA) components. Int. J. Adv. Manuf. Technol..

[11] Tunçel O., Tüfekci K., Kahya Ç. (2024). Multi-objective optimization of 3D printing process parameters using gray-based Taguchi for composite PLA parts. Polym. Compos..

[12] Özen A., Abali B.E., Völlmecke C., Gerstel J., Auhl D. (2021). Exploring the role of manufacturing parameters on microstructure and mechanical properties in fused deposition modeling (FDM) using PETG. Appl. Compos. Mater..

[13] Kopar M., Erdaş M.U., Yıldız A.R. (2024). Experimental Investigation on Mechanical properties of CF15PET and GF30PP materials produced with different raster angles. Mater. Test..

[14] Economides A.L., Islam M.N., Baxevanakis K.P. (2024). Additively manufactured carbon fibre PETG composites: Effect of print parameters on mechanical properties. Polymers.

[15] Prajapati S., Sharma J.K., Kumar S., Pandey S., Pandey M.K. (2024). A review on comparison of physical and mechanical properties of PLA, ABS, TPU, and PETG manufactured engineering components by using fused deposition modelling. Mater. Today Proc..

[16] Özen A., Auhl D., Völlmecke C., Kiendl J., Abali B.E. (2021). Optimization of Manufacturing Parameters and Tensile Specimen Geometry for Fused Deposition Modeling (FDM) 3D-Printed PETG. Materials.

[17] Issametova M., Martyushev N.V., Zhastalap A., Sabirova L.B., Assemgul U., Tursynbayeva A., Abilezova G. (2024). Determination of residual stresses in 3D-printed polymer parts. Polymers.

[18] Kim S., Kim E.H., Lee W., Sim M., Kim I., Noh J., Kim J.H., Lee S., Park I., Su P.C. (2024). Real-time in-process control methods of process parameters for additive manufacturing. J. Manuf. Syst..

[19] Viano R., Demont L., Margerit P., Mesnil R., Caron J.F., Weisz-Patrault D. (2025). Residual stress control in large-format additive manufacturing of polylactic acid via a digital twin and in-operando imaging. Mater. Des..

[20] Lim A., Pathak P., Selvamanickam V., Hernandez F.C.R. (2025). In-Situ/In-Operando Approach for Topology Optimization of Structural Components by Means of Thermal Imaging, Thermal Analysis and CT Scan. J. Jpn. Soc. Powder Powder Metall..

[21] Uspensky B., Derevianko I., Avramov K., Maksymenko-Sheiko K., Chernobryvko M. (2025). Mechanical properties of auxetic honeycombs realized via material extrusion additive manufacturing: Experimental testing and numerical studies. Appl. Compos. Mater..

[22] Harley W.S., Vidler C., Tee Y.L., Kolesnik K., Heath D.E., Tran P., Collins D.J. (2025). 3D printing of TPMS microlattice: Indentation characterisation and numerical analysis. Virtual Phys. Prototyp..

[23] Shin W., Han J. (2024). Multiscale analysis for characterization of tensile properties in extrusion-based 3D printed carbon nanocomposites. Addit. Manuf..

[24] Liu P., Qi W., Luo K., Yin C., Li J., Lu C., Lu L. (2024). Bending performance and failure mechanisms of composite sandwich structures with 3D printed hybrid triply periodic minimal surface cores. J. Sandw. Struct. Mater..

[25] Di Michele F., Styahar A., Pera D., Aloisio R., Rubino B., Marcati P. (2024). Shape effects on wave propagation in a 2D domain using the finite element method. Math. Mech. Complex Syst..

[26] Rosi G., Auffray N., Combescure C. (2024). Elastic wave propagation in cubic non-centrosymmetric and chiral architectured materials: Insights from strain gradient elasticity. Int. J. Solids Struct..

[27] Eskandari S., Xu W., Chen S., Abali B.E., Orsat V., Akbarzadeh A. (2025). Elastic-Wave Propagation in Chiral Metamaterials: A Couple-Stress Theory Perspective. Adv. Eng. Mater..

[28] Abdoul-Anziz H., Seppecher P. (2018). Strain gradient and generalized continua obtained by homogenizing frame lattices. Math. Mech. Complex Syst..

[29] Alibert J.J., Barchiesi E., Dell’isola F., Seppecher P. (2023). A class of one dimensional periodic microstructures exhibiting effective Timoshenko beam behavior. ESAIM Control Optim. Calc. Var..

[30] Hornỳ L., Petřivỳ Z., Sobotka Z., Kohan M., Balint T., Chlup H., Kronek J., Mendová K., Hudák R., Schnitzer M. (2025). Notes on constitutive modeling of 3D-printed PLA materials. J. Mech. Behav. Biomed. Mater..

[31] Abali B.E., Barchiesi E. (2021). Additive manufacturing introduced substructure and computational determination of metamaterials parameters by means of the asymptotic homogenization. Contin. Mech. Thermodyn..

[32] Fedele R., Placidi L., Fabbrocino F. (2024). A review of inverse problems for generalized elastic media: Formulations, experiments, synthesis. Contin. Mech. Thermodyn..

[33] Terranova L.M., Turco E., Misra A., dell’Isola F. (2025). Computational identification of double-bending stiffness: From Zigzagged Articulated Parallelograms with Articulated Braces (ZAPAB) structures to pure-curvature gradient planar inextensible 1D continua. Comptes Rendus Mécanique.

[34] Akhoundi B., Behravesh A.H. (2019). Effect of filling pattern on the tensile and flexural mechanical properties of FDM 3D printed products. Exp. Mech..

[35] Afshar R., Jeanne S., Abali B.E. (2023). Nonlinear Material Modeling for Mechanical Characterization of 3-D Printed PLA Polymer With Different Infill Densities. Appl. Compos. Mater..

[36] Bhuiyan M.Z.H., Khanafer K., Rafi E.I., Shihab M.S. (2025). Non-Linear hyperelastic model analysis and numerical validation of 3D printed PLA+ material incorporating various infill densities. Machines.

[37] Abali B.E., Afshar R., Khaksar N., Segersten D., Vedin T. (2024). Damage Behavior in Additive Manufacturing based on Infill Pattern and Density with Carbon Particle Filled PolyLactic Acid (CF-PLA) Polymer Filaments. State of the Art and Future Trends in Materials Modelling 2.

[38] Mukherjee T., Kao N. (2011). PLA based biopolymer reinforced with natural fibre: A review. J. Polym. Environ..

[39] Niu G., Tao Y., Zhang R., Feng X., Deng H., Ren M., Sun J., Peijs T. (2024). Toughening of self-reinforced PLA films using PLA nanofiber mats and oriented PLA tapes as interlayers. Compos. Part A Appl. Sci. Manuf..

[40] Yadav N., Richter T., Löschke O., Abali B.E., Auhl D., Völlmecke C. (2023). Towards Self-Reinforced PLA Composites for Fused Filament Fabrication. Appl. Sci..

[41] Afshar R., Jeanne S., Abali B.E., Altenbach H., Berezovski A., dell’Isola F., Porubov A. (2023). Effects of 3-D Printing Infill Density Parameter on the Mechanical Properties of PLA Polymer. Sixty Shades of Generalized Continua: Dedicated to the 60th Birthday of Prof. Victor A. Eremeyev.

[42] Patel S.K., Gupta S., Saket H., Bakna K., Patel S.S., Kumar S., Ramakoteswara Rao V., Mandava R.K. (2024). Effect of infill pattern on the mechanical properties of PLA and ABS specimens prepared by FDM 3D printing. Proc. Inst. Mech. Eng. Part E J. Process Mech. Eng..

[43] Yang Y., Wang Z., He Q., Li X., Lu G., Jiang L., Zeng Y., Bethers B., Jin J., Lin S. (2022). 3D Printing of Nacre-Inspired Structures with Exceptional Mechanical and Flame-Retardant Properties. Research.

[44] Patadiya J., Wang X., Joshi G., Kandasubramanian B., Naebe M. (2023). 3D-Printed Biomimetic Hierarchical Nacre Architecture: Fracture Behavior and Analysis. ACS Omega.

[45] Curto M., Dowsett J., Kao A.P., Tozzi G., Barber A.H. (2025). Multi-material 3D printed composites inspired by nacre: A hard/soft mechanical interplay. Sci. Rep..

[46] Lv X., Wang S., Xu Z., Liu X., Liu G., Cao F., Ma Y. (2023). Structural Mechanical Properties of 3D Printing Biomimetic Bone Replacement Materials. Biomimetics.

[47] Song X., Hong S., Wang J., Zhu X., Guo S., Fu Y., Yang Y., Yang M., He W., Tang Y. (2024). Mechanical Properties of a Honeycomb Structure Dispersed with 3D-Printed Fe_3_O_4_ Nanomaterials. ACS Omega.

[48] Dong X., Zhang R., Tian Y., Ramos M.A., Hu T.S., Wang Z., Zhao H., Zhang L., Wan Y., Xia Z. (2020). Functionally Graded Gecko Setae and the Biomimics with Robust Adhesion and Durability. ACS Appl. Polym. Mater..

[49] Trink N., Magdassi S. (2024). Tunable Lotus Leaf Effect by Three-Dimensionally Printed Stretchable Objects. ACS Appl. Mater. Interfaces.

[50] Inoue M., Saito K., Aoyama H., Inoue H., Ohnuki R., Yoshioka S. (2025). Study on the chirality of gyroid photonic crystals in butterfly wing scales. Sci. Rep..

[51] Nazzi F. (2016). The hexagonal shape of the honeycomb cells depends on the construction behavior of bees. Sci. Rep..

[52] Marinković S., Stanković P., Štrbac M., Tomić I., Ćetković M. (2012). Cochlea and other spiral forms in nature and art. Am. J. Otolaryngol..

[53] (2004). Standard Test Method for Moisture Absorption Properties and Equilibrium Conditioning of Polymer Matrix Composite Materials.

[54] (2019). Plastics—Determination of Flexural Properties.

[55] Aydin G., Sarar B.C., Yildizdag M.E., Abali B.E. (2022). Investigating infill density and pattern effects in additive manufacturing by characterizing metamaterials along the strain-gradient theory. Math. Mech. Solids.

[56] Ashby M.F., Shercliff H., Cebon D. (2018). Materials: Engineering, Science, Processing and Design.

[57] Álvarez-Blanco M., Abali B.E., Völlmecke C. (2025). An experimental methodology to determine damage mechanics parameters for phase-field approach simulations using material extrusion-based additively manufactured tensile specimens. Virtual Phys. Prototyp..

[58] Atakok G., Kam M., Koc H.B. (2022). Tensile, three-point bending and impact strength of 3D printed parts using PLA and recycled PLA filaments: A statistical investigation. J. Mater. Res. Technol..

